# Cancer—A Major Cardiac Comorbidity With Implications on Cardiovascular Metabolism

**DOI:** 10.3389/fphys.2021.729713

**Published:** 2021-11-26

**Authors:** Daniel Finke, Markus B. Heckmann, Norbert Frey, Lorenz H. Lehmann

**Affiliations:** ^1^Cardio-Oncology Unit, University Hospital Heidelberg, Heidelberg, Germany; ^2^Department of Cardiology, University Hospital Heidelberg, Heidelberg, Germany; ^3^DZHK (German Centre for Cardiovascular Research), Partner Site Heidelberg/Mannheim, Heidelberg, Germany; ^4^Deutsches Krebsfoschungszentrum (DKFZ), Heidelberg, Germany

**Keywords:** cardio-oncology, cancer metabolism, cardiac metabolism, cytokines, second messenger, metabolic shift, inflammation, heart failure

## Abstract

Cardiovascular diseases have multifactorial causes. Classical cardiovascular risk factors, such as arterial hypertension, smoking, hyperlipidemia, and diabetes associate with the development of vascular stenoses and coronary heart disease. Further comorbidities and its impact on cardiovascular metabolism have gotten more attention recently. Thus, also cancer biology may affect the heart, apart from cardiotoxic side effects of chemotherapies. Cancer is a systemic disease which primarily leads to metabolic alterations within the tumor. An emerging number of preclinical and clinical studies focuses on the interaction between cancer and a maladaptive crosstalk to the heart. Cachexia and sarcopenia can have dramatic consequences for many organ functions, including cardiac wasting and heart failure. These complications significantly increase mortality and morbidity of heart failure and cancer patients. There are concurrent metabolic changes in fatty acid oxidation (FAO) and glucose utilization in heart failure as well as in cancer, involving central molecular regulators, such as PGC-1α. Further, specific inflammatory cytokines (IL-1β, IL-6, TNF-α, INF-β), non-inflammatory cytokines (myostatin, SerpinA3, Ataxin-10) and circulating metabolites (D2-HG) may mediate a direct and maladaptive crosstalk of both diseases. Additionally, cancer therapies, such as anthracyclines and angiogenesis inhibitors target common metabolic mechanisms in cardiomyocytes and malignant cells. This review focuses on cardiovascular, cancerous, and cancer therapy-associated alterations on the systemic and cardiac metabolic state.

## Introduction

Currently, a percentage of 18% of cancer patients are seen with cardiovascular comorbidities in large population-based studies. The highest rates are observed in prostate cancer patients (36.5%) or patients with endometrial carcinoma (29.2%). Cardiovascular complications range from stroke to arrhythmias, coronary heart disease (CHD), cardiomyopathy, heart failure (HF), or valvular diseases (Liu et al., 2019). Death due to cardiovascular complications is mostly seen in patients suffering from prostate cancer, breast cancer, lung cancer or urinary bladder cancer (Sturgeon et al., 2019).

Cardiovascular diseases itself, such as CHD and HF, are mostly attributed to the occurrence of cardiovascular risk factors (e.g., arterial hypertension, diabetes, smoking, age, hypercholesterinemia, and adiposity) or predisposing genetic factors (Jacoby and McKenna, 2012; McPherson and Tybjaerg-Hansen, 2016; Piepoli et al., 2016).

These are not solely risk factors for cardiovascular diseases, but also increase the risk of cancer (Freedman et al., 2011; Stocks et al., 2012; Koene et al., 2016; Lauby-Secretan et al., 2016). Recently, the interdisciplinary field of cardio-oncology focuses on the interaction of both diseases in terms of the shared risk profile, but also in terms of a direct interaction between the two systemic diseases (Cardinale, 1996).

Commonly known are adverse effects of cancer therapy (Tilemann et al., 2018). Therefore, the cardiotoxic cardiomyopathy is the initial interdisciplinary focus of cardio-oncology (Cardinale et al., 2015; Rassaf et al., 2020). The field shifts to a paradigm of malignant diseases itself as a risk factor for the development of cardiovascular complications and vice versa (Demers et al., 2012; Gernaat et al., 2018). Currently, efforts are made to describe the differential role of HF and CHD in the progression of cancer (Rinde et al., 2017; Koelwyn et al., 2020; Lau et al., 2021).

The molecular interaction of heart disease and cancer have multiple layers. The development of inflammatory processes, either of cardiac or malignant origin, is one factor (Anker and von Haehling, 2004; Frantz and Nahrendorf, 2014; Noy and Pollard, 2014; Duan and Luo, 2021). Increased oxidative stress is another mechanism which is discussed to connect both diseases (Kanai et al., 2001; Chan et al., 2017). Additionally, different cytokines and paracrine mechanisms mediate a direct crosstalk (Quail and Joyce, 2013; Bang et al., 2015; Olson et al., 2017).

Besides, metabolic disorders are linked to cancer and HF (Karlstaedt et al., 2018). In fact, a reprogrammed metabolism is essential for tumor growth, including an increased uptake of glucose and amino acids, different use of metabolic intermediates and alterations in gene expression or microenvironmental interactions (Pavlova and Thompson, 2016). Anaerobic glycolysis and lactate production is one of the central mechanisms in tumor metabolism. This includes a dysregulation of key enzymes in different glycolysis pathways.

In heart disease, various metabolic adaptations, such as switch from lipid to glucose metabolism in HF (Murashige et al., 2020) and diabetic cardiomyopathy, are associated with an increased myocardial stiffness (van Heerebeek et al., 2008) and altered Ca^2 +^ -handling (Lambert et al., 2015; Seferovic and Paulus, 2015). There are concurrent adaptations in glucose metabolism, including glycolysis and its side pathways, in cancer and HF.

Nevertheless, the direct interaction of cardiac and cancer metabolism is an emerging field which needs further evaluation. The oncometabolite D-2-hydroxyglutarate (D2-HG) is one example of a primary metabolic interaction of cancer and the heart (Karlstaedt et al., 2016; Kattih et al., 2021).

This review focuses on the available data of concurrent and causative metabolic events in cardiac disease and cancer and its impact on cardiac dysfunction and cancer progression, respectively.

## Cancer Therapies and Cancer Itself May Influence the Cardiac Function

Initially, the cardiotoxic effects of chemotherapies are seen as the primary connection between cardiac function and cancer. Especially, a drop in left ventricular function is associated with the administration of anthracyclines (Zambetti et al., 2001). The majority of studies that deal with cardiotoxicity has concentrated on breast cancer patients (Levis et al., 2017). This may be due to the broad usage of cardiotoxic anthracyclines but also to a favorable outcome with the possibility of larger observation intervals. Later, also further drugs, such as trastuzumab, were linked to cardiotoxicity (Slamon et al., 2001; Ozcelik et al., 2002).

Cardiotoxicity is initially defined as a declined left ventricular function. More recent studies describe changes in diastolic function or cardiac deformation (strain measurements) (Bird and Swain, 2008; Serrano et al., 2015). Also in patient cohorts that are not restricted to breast cancer patients, cardiac biomarkers (including N-terminal pro B-type natriuretic peptide (NT-proBNP), mid-regional pro-atrial natriuretic peptide (MR-proNAP), mid-regional pro-adrenomedullin (MR-proADM), C-terminal pro-endothelin-1 (CT-pro-ET1), and high sensitivity cardiac troponin T (hs-cTnT)) are found to be associated with cancer and cancer therapies. In cardiac disease, the natriuretic peptides are generally parameters for heart failure and pressure overload, hs-cTnT is primarily related to cardiac cell death (Pavo et al., 2015). Patients with elevated levels of NT-proBNP and hs-cTnT often show a preserved ejection fraction (Finke et al., 2021).

Apart from these observations, no studies have dissected HF with reduced (HFrEF) and preserved ejection fraction (HFpEF) in terms of chemotherapies or the neoplastic disease itself. A recent study, the CARDIOTOX registry, suggests a new definition of cardiotoxicity. Here, the systolic function and cardiac biomarkers are taken into account, neither including the diastolic function nor strain measurements (Lopez-Sendon et al., 2020).

Beyond the effects of chemotherapies, there is a direct crosstalk between cancer and the heart. On the one hand, HF may lead to incident cancer and vice versa cancer itself may lead to cardiac dysfunction (Meijers and de Boer, 2019). A large population-based analysis of patients with myocardial infarction (MI) for example finds that patients with MI had higher incidences of cancer than comparable patients without MI. The highest rates are observed for colorectal cancer, prostatic cancer and lung cancer (Rinde et al., 2017). In addition, cancer patients are reported to have higher rates of cardiac complications (Sturgeon et al., 2019). If those complications are based on the shared risk profiles, treatment-related effects or a direct interaction of the diseases is frankly difficult to dissect in a clinical setting. Molecular studies focus on specific alterations due to cancer itself and link them with adverse cardiac effects. Among those alterations, secreted and circulating molecules are found, which may directly affect cardiac function. These molecules are an independent risk factor of cardiac dysfunction in cancer patients, apart from cancer therapies (see Table 1).

**TABLE 1:** Preclinical models that describe metabolic alterations in heart disease, cancer, and link both diseases (interdisciplinary studies).

**Factor**	**Metabolic disorder**	**Intervention**	**Cardiovascular studies**	**Cancer studies**	**Interdisciplinary studies**
**Inflammation**					
IFN-γ	Cachexia	Anti-IFN-γ antibodies		Matthys et al., 1991a	
IL-6	Cachexia	IL-6 KO animals		Cahlin et al., 2000	
	Cachexia	Anti-IL-6 antibodies		Strassmann et al., 1992	
	Muscular atrophy	IL-6 transgenic mice			Tsujinaka et al., 1995
	Muscular atrophy	IL-6 injections			Haddad et al., 2005
TNF-α	Cachexia	Injection of TNF-α -producing CHO cells		Oliff et al., 1987	
	Cachexia	Pharmacological inhibition of TNF-α	Steffen et al., 2008		
IL-1β	Cachexia	Injection of IL1-receptor antagonists		Strassmann et al., 1993	
**TGF-β signaling**	Cachexia and loss of heart mass	Blockage of Activin ActRIIB pathway in C26 tumor-bearing mice			Zhou et al., 2010
**Myokines**					
Myostatin	Muscle wasting	Cardiomyocyte-specific deletion of Myostatin and use of a myostatin blocking antibody	Heineke et al., 2010		
SerpinA3	Tumor growth	MI/HTx in APC^min^ mice			Meijers et al., 2018
**Cachexokines**					
Ataxin-10	Cardiac atrophy	Tumor cell (MC38, C26, SW480) injection in mice, APC delta 580 mice			Schafer et al., 2016
Insulin depletion	Cardiac wasting	Insulin supplementation			Thackeray et al., 2017
**MicroRNAs**					
miR-145	Cardiac fibrosis	miR-145 KO mice	Zhao et al., 2015		
	Tumor growth	miR/miR inhibitor transfection		Xing et al., 2013	
		miR transfection		Xia et al., 2014	
miR-1	Elevated in MI after LAD ligation	LAD ligation	D’Alessandra et al., 2010		
	Tumor growth suppression	miR transfection		Liu et al., 2017	
miR-133a/b	Elevated in MI after LAD ligation	LAD ligation	D’Alessandra et al., 2010		
	Heart malformations/DCM	miR-133a-1/2 dKO mice	Liu et al., 2008		
	Tumor growth suppression	miR transfection		Qin et al., 2013	
miR-208a	AV blockage/cardiac hypertrophy	mR-208a Tg/KO mice	Callis et al., 2009		
	Oncogene/Tumor proliferation	miR overexpression/miR siRNA			Li et al., 2014
**Metabolism**					
Akt	Heart failure and mitochondrial dysfunction	Akt transgenic mice, constative active and inducible	Wende et al., 2015		
	Enhanced glucose metabolism, GLUT-1 expression	Inducible Akt-transgenic mouse hepatoma cells		Barthel et al., 1999	
	HK activity, HK-mitochondria interaction	Akt-1 KO, Akt-1/2 dKO		Majewski et al., 2004	
PGC-1α	Heart failure	PGC-1α KO mice	Arany et al., 2006		
	PPCM	PGC-1α KO mice	Patten et al., 2012		
	Apoptosis	Pancreatic Cancer Stem Cells/Metformin/MYC expression		Sancho et al., 2015	
	Glycolytic flux and poor prognosis in breast cancer	Breast cancer cell lines/siRNA treatment		McGuirk et al., 2013	
O-GlcNAc	Cardiac Hypertrophy	Db/db mice	Marsh et al., 2011		
	Heart failure	Db/db mice, STZ injections, HDAC4 KO mice	Kronlage et al., 2019		
	Transient CMP	HDAC4 KO mice, STIM1/2 dKO mice	Lehmann et al., 2018		
	Glycolytic flux/apoptosis	Breast cancer cell line/OGT-shRNA transfections		Ferrer et al., 2014	
		Pancreas duct epithelial cells/OGT-shRNA transfections		Ma et al., 2013	
	Tumor growth	Xenograft models with shOGT		Caldwell et al., 2010	
**Circulating metabolites**					
D2-HG	Alterations in cardiac glucose utilization and epigenetic repression	IDH2^R140Q^-mutant mice			Karlstaedt et al., 2016
	Cardiotoxicity	AML patients with IDH1/2, iPS derived cardiomyocytes			Kattih et al., 2021

*ActRIIB, Activin type 2 receptor; APC, Adenomatosis polyposis coli; AV, Atrioventricular; CHD, Coronary heart disease; CMP, Cardiomyopathy; CHO-cells, Chinese Hamster Ovarian cells; D2-HG, D-2-Hydroxyglutarate; DCM, Dilatative cardiomyopathy; dKO, Double Knockout; GLUT-1, Glucose transporter 1; HTx, Heart transplantation; HK, Hexokinase; HDAC4, Histon deacetylase 4; IFN-γ, Interferon-gamma; IL, Interleukin; IDH2, Isocitrate dehydrogenase 2; KO, Knockout; LAD, Left anterior descending coronary artery; Db/Db, Leptin receptor-deficient mouse line; miR, MicroRNA; Min, Multiple intestinal neoplasia; MI, Myocardial infarction; O-GlcNAc, O-Linked N-Acetylglucosamine; PPCM, Peripartum cardiomyopathy; PGC-1α, Peroxisome proliferator-activated receptor-gamma coactivator; OGT, Protein O-GlcNAc Transferase; Akt, Protein kinase B; shRNA, small hairpin RNA; siRNA, Small interfering RNA; STZ, Streptozotocin; STIM1/2, Stromal interaction molecule 1/2; TNF-α, Tumor necrosis factor alpha; Tg, Transgene; TGF-β, Tumor growth factor beta.*

## Cancer—A Systemic Disease—Affects the Heart

Cancer is a *debilitating* disease. In many cases, cancer leads to cachexia based on alterations of metabolic pathways. Clinically, it results in fatigue, frailty and a reduced quality of life. Finally, the mortality rates of cancer patients are even higher due to cachexia (von Haehling and Anker, 2014).

One manifestation of cachexia is skeletal muscle wasting, which is one of the main causes for the mentioned detrimental effects. The loss of muscle causes serious metabolic alterations. In skeletal muscles, high rates of protein degradation result in amino acid accumulation. These amino acids serve as substrates for gluconeogenesis in the liver. The elevated glucose levels and glutamine, a released amino acid from the muscles, serve as energy supply for the neoplastic tissues and basis for protein or DNA synthesis (Cluntun et al., 2017). The high energy demand of the tumor results in high levels of circulating lactate, which in turn is used for gluconeogenesis in the liver. This is the basis for the energy-inefficient Cori cycle. Adipose tissue is wasted during this process. Triacylglycerols (TAGs) are lipolyzed to glycerol and non-essential fatty-acids (NEFAs), which again are used for gluconeogenesis and energy supply for the tumor, respectively (Argiles et al., 2014).

Preclinical data indicates that cardiac muscle wasting is associated with cancer cachexia. In a hepatoma rat model, the authors observe left ventricular (LV) dysfunction, fibrotic cardiac remodeling and increased mortality (Springer et al., 2014). Accordingly, in patients with tumor cachexia, bodyweight loss and muscular atrophy is associated with loss of cardiac weight (Barkhudaryan et al., 2017). Patients with Hodgkin lymphoma show an increase in cardiac glucose uptake, supporting the hypothesis of a direct metabolic re-programming in certain entities of cancer (Heckmann et al., 2019). In turn, HF is associated with loss of lean tissue mass and reduced exercise capacity, also because of a reduction of skeletal muscle mass (Strassburg et al., 2005). In addition, wasting is identified as an independent risk factor for mortality in HF patients (Anker et al., 1997b).

Trying to unravel the pathophysiology of wasting and its correlation with the cardiac function, several mechanisms have been identified. Malnutrition and malabsorption from the gut, inflammation and hormonal regulations are some mediating factors (von Haehling et al., 2007). All of these may be influenced by cancer and HF and vice versa.

## Malnutrition in Heart Failure and Cancer

Cancer-associated malnutrition has multiple reasons. On the one hand, there are adverse effects of cancer treatment and local, obstructive effects of the tumor itself, which lead to a reduced food intake. On the other hand, HF may lead to bowel edema and impairments in gut perfusion. This, in turn, results in malabsorption as well (King et al., 1996).

The gut microbiota is changed in HF patients, too (Tang et al., 2019). Pathogenic gut flora overgrowth, including Candida, Campylobacter, Shigella, Salmonella, and Yersinia enterocolitica, is present in HF patients in an observational study from Pasini et al. (2016). The authors are able to link the pathogenic populations to the severity of HF using the clinical New York Heart Association classification (Pasini et al., 2016).

Splanchnic congestion may lead to impaired intestinal barrier function and thereby to a systemic inflammatory response and cytokine release, which in turn may result in lean mass loss and cardiac muscle wasting (Aoyagi et al., 2015). Due to malabsorption and malnutrition, Cluster of differentiation 14 (CD14) levels are elevated in cachectic HF patients in line with increased levels of tumor necrosis factor alpha (TNF-α), indicating an endotoxin release with a concomitant inflammatory response (Anker et al., 1997a).

## The Role of an Inflammatory Response, Originated From the Heart or Cancer, and Its Implication in Metabolic Disorders

The levels of inflammatory circulating factors, such as soluble tumor necrosis factor receptors (sTNF-Rs), soluble intercellular adhesion molecule-1 (sICAM-1), interleukine (IL)-6, lipopolysaccharide binding protein (LBP), and C-reactive protein (CRP) are associated with weight loss in patients with non-small-cell lung cancer (NSCLC) (Staal-van den Brekel et al., 1995). Elevated CRP levels and weight loss are seen in patients with a reduced physical function and prognosis. Weight loss as a single factor does not identify the patients at risk sufficiently (Fearon et al., 2006). Preclinical models are used to further understand the pathomechanism of an inflammatory response in cancer or the heart and its effect on metabolism.

Interferon-gamma (IFN-γ) producing Chinese Hamster Ovary cells (CHO-cells) are injected in mice and cause cachexia solely in the presence of IFN-γ and tumor cells. Inhibition of IFN-γ or the injection of non-IFN-γ producing cells do not result in a comparable degree of cachexia (Matthys et al., 1991a). In an animal model of lung cancer, IFN-γ is necessary for the development of tumor cachexia and is blocked by administration of anti-IFN-γ antibodies (Matthys et al., 1991b). Whether this is associated with an atrophy of cardiac muscle has not been investigated.

IFN-γ knockout (KO) mice do not show mitigated response to tumor-induced cachexia in a model of sarcoma in C57BL6 mice. This may indicate a tumor- and non-host-dependent mechanism of IFN-γ in cachexia. In turn, IL-6 KO animals show no tumor-associated cachexia (Cahlin et al., 2000). Anti-IL-6 antibodies are able to mitigate tumor cachexia in a mouse model of colon cancer (Strassmann et al., 1992). IL-6 transgenic mice show muscular atrophy of the gastrocnemius muscle (Tsujinaka et al., 1995). Mechanistically, IL-6 injections are linked to phosphorylation and thereby to activation of the transcription factor (TF) signal transducer and activator of transcription 3 (STAT3) (Haddad et al., 2005; Bonetto et al., 2012).

TNF-α, another proinflammatory cytokine, is also found to regulate muscle wasting. Mice which are treated with TNF-α secreting CHO cells show a similar phenotype, including cachexia and a poor outcome compared to IL-6 treated animals (Oliff et al., 1987). Pharmacological inhibition of TNF-α is able to attenuate cardiac cachexia in a rat model (Steffen et al., 2008). In response to TNF-α, induced activation of NFκB is responsible for ubiquitin/proteasome activity. Muscle-specific proteins, such as myosin heavy chain, are thereby degraded (Li et al., 1998; Li and Reid, 2000).

IL-1β is shown to have a similar effect on cancer-associated cachexia (Laird et al., 2021). By use of IL1-receptor antagonists tumor cachexia, as well as growth of colon cancer in C57BL6 mice, are attenuated (Strassmann et al., 1993).

Apart from inflammation, other factors are shown to be linked to tumor cachexia in preclinical studies. The blockage of the transforming growth factor-beta (TGF-β) signaling pathway is able to ameliorate cachexia and survival in tumor-bearing mice, independent of inflammatory cytokines. Additionally, the authors are able to link the positive effects of prevention of cachexia to reduced cardiac wasting (Zhou et al., 2010).

## Second Messengers Mediate Metabolic Changes

A direct link between cancer and cardiac function is found *via* circulating molecules. The heart as well as cancer cells release these factors or are affected by them, vice versa.

By cytokine release, the heart may function as an endocrine organ. Several mediators of cardiac origin have been found, which have an impact on metabolic regulations and tumor growth.

Myostatin, for example, is found to be released from the heart under failing conditions. This in turn promotes an inhibitory effect on muscle growth and may lead to muscle wasting. In cardiomyocyte-specific myostatin KO animals, wasting of the periphery muscles is attenuated after pressure-overload (Heineke et al., 2010).

Other factors—such as SerpinA1/3, Fibronectin, Ceruloplasmin, and Paraxonase 1—that are secreted from the heart, are found to be elevated in HF. Regarding SerpinA3, a direct influence on cancer cell growth is found. SerpinA3 is able to induce growth of colon cells and activates the protein kinase B signaling (Akt) pathway (Meijers et al., 2018). Akt signaling is essential in mediating glucose uptake and may inhibit pro-apoptotic factors (Nitulescu et al., 2018).

However, neoplastic cells in turn can release factors that affect metabolism and cardiac function. Insulin depletion, which can be mediated by tumorous diseases, causes a decreased glucose uptake of the heart (a sign for a pathological metabolic switch) and is thereby a reason for cardiac muscle wasting. Insulin supplementation is able to attenuate the adverse effects on the heart (Thackeray et al., 2017). Ataxin-10 is another cachexokine, that has been identified by an unbiased approach, which investigates the pro-atrophic secreted proteins of a certain colon cancer entity. Ataxin-10 causes cardiac metabolic alterations and muscle wasting (Schafer et al., 2016).

Recently, Karlstaedt et al. (2016) have found evidence for a metabolic messenger which is derived from cancer cells and is able to affect the cardiac metabolism, respectively. The authors characterize a mouse model for acute myeloid leukemia with an IDH2 mutation. The excreted metabolite D2-HG, which is derived from the leukemic cells, alters glucose utilization and histone modifications in the heart. They mainly attribute these effects to an inhibition of the α-ketoglutarate dehydrogenase activity by D2-HG in the heart. These alterations are associated with cardiac remodeling (Karlstaedt et al., 2016). In cell culture, the oncometabolite 2-HG exacerbates doxorubicin mediates cardiotoxicity (Kattih et al., 2021).

There are still only few studies, investigating metabolomic alterations in patients plasma before, during or after oncological disease (Schmidt et al., 2021). However, in certain entities (e.g., pancreatic cancer) alterations of the metabolome are strongly associated with an increased risk to develop cancer (Mayers et al., 2014). If these alterations associate with an increased risk for cardiac complications, needs further evaluation.

Second messengers that may alter cancer and heart metabolism can also be of other origins. Myokines are cytokines which are released from the muscle (Manole et al., 2018). In cancer-induced cachexia, the levels of several myokines—including myostatin, IL-15, follistatin-related protein 1 (FSTL-1), fatty acid binding protein 3 (FABP3) and irisin—are elevated, suggesting that inhibition of myokines could be a therapeutical approach (de Castro et al., 2021).

Another group of circulating factors which influence cancer and the heart are microRNAs (miRs). MiR-145, for example, is found to be involved in the growth of many different tumors. It mainly functions as a tumor suppressor (Xing et al., 2013; Xia et al., 2014). MiR-145 also suppresses pathological remodeling in cardiovascular disease. Zhao et al. (2015) are able to link miR-145 to TGF-β signaling and to suppression of Angiotensin II-induced cardiac fibrosis. Other miRs, such as miR-1, miR-133, and miR-208, have been linked with cancer and cardiovascular diseases (Fichtlscherer et al., 2011; Mitchelson and Qin, 2015). Those miRs are elevated in the serum after ligation of the left anterior descending coronary artery in mice and after acute coronary syndrome (ACS) in patients (D’Alessandra et al., 2010; De Rosa et al., 2011). MiR-133a-1/2 dKO mice die due to developmental heart defects or, if they survive for at least six months, suffer from dilated cardiomyopathy. MiR-133-deficiency is linked to increased expression of the transcription factor (TF) serum response factor (SRF) (Liu et al., 2008). MiR-208a transgenic mice show alterations in the cardiac conduction system, e.g., atrioventricular blocks (Callis et al., 2009). In tumor cell lines, miR-1 and miR-133 are found to be tumor-suppressive, miR-208 in turn promotes tumor growth (Qin et al., 2013; Li et al., 2014; Liu et al., 2017).

## Comparing the Metabolic Shift in Heart Failure and Cancer

Apart from studies of circulating metabolic factors that directly link cancer and the heart, comparable alterations in metabolism have been described in cardiac disease and cancer. These changes are summarized in Table 2.

**TABLE 2:** Regulation of metabolic genes in failing cardiomyocytes and cancer cells.

**Gene**	**Failing cardiomyocyte**	**Cancer cell**
Akt	• Akt is upregulated in the heart due to pressure-overload (Wende et al., 2015) • Repression of FAO (Wende et al., 2015)	• Akt pathway activation is leading to cell growth (Sancho et al., 2015) • Akt enhances glucose supply *via* GLUT-1 and HK1 upregulation (Barthel et al., 1999; Majewski et al., 2004)
PGC-1α	• Downregulation in heart failure (Arany et al., 2006) • Inhibition deteriorates heart function during pressure-overload (Arany et al., 2006)	• Upregulation in breast cancer increases glutamine flux and glycolysis rates (McGuirk et al., 2013) • PGC-1α positively regulates cellular respiration in the mitochondrium (Wu et al., 1999)
OGT/OGA (O-GlcNAc)	• Increased O-GlcNAc is protective in the diabetic heart (Marsh et al., 2011; Kronlage et al., 2019) • Inhibition of O-GlcNAc protects from pressure-overload (Umapathi et al., 2021)	• Decreased O-GlcNAc induces apoptosis and reduces growth of breast cancer and pancreatic tumors (Caldwell et al., 2010; Ma et al., 2013; Ferrer et al., 2014)
PFK	• Inhibition deteriorates heart function during pressure-overload (Wang et al., 2013)	• Upregulation in cancer and maintenance of a high glycolytic flux, e.g., in leukemia cells (Chesney et al., 1999)

*FAO, Fatty acid oxidation; GLUT-1, Glucose transporter 1; HK, Hexokinase; PFK, Phosphofructokinase; PGC-1α, Peroxisome proliferator-activated receptor-gamma coactivator; Akt, Protein kinase B; O-GlcNAc, O-Linked N-Acetylglucosamine; OGT, Protein O-GlcNAc Transferase; OGA, protein O-GlcNAcase; TopoIIβ, Topoisomerase IIβ; VEGF, Vascular endothelial growth factor.*

In HF, there is a shift in metabolic substrate utilization to glucose. In the normal heart, ATP-production mainly relies on fatty acid oxidation (FAO). This phenomenon is termed metabolic remodeling (Tran and Wang, 2019).

Genetic deletion of RBPJ, a regulator of the NOTCH signaling pathway, leads to the disability of endothelial cells for fatty acid uptake in the muscle and heart. Consequently, FDG-PET/CT show a compensatory uptake of glucose in the heart. Mice that are deficient of RBPJ suffer from a reduced systolic ejection fraction, in line with elevated glucose-6-phosphat levels. A ketogenic diet is able to rescue this phenotype (Jabs et al., 2018). Metabolic remodeling is therefore not only a consequence of cardiac dysfunction but also causatively involved.

In pathological cardiac hypertrophy, due to pressure overload, the concurrent Akt signaling is mediating a decline in mitochondrial function and repression of FAO (Wende et al., 2015). Peroxisome proliferator-activated receptor-gamma coactivator (PGC-1α) regulates FAO and proteins involved in the electron transport chain. These factors are downregulated in HF. PGC-1α deficiency aggravates this process in response to pressure overload. Subsequently, the transition to HF is developing more rapidly (Arany et al., 2006). In LV hypertrophy, however, glycolysis rates are increased (Nascimben et al., 2004). Inhibiting phosphofructokinase (PFK), the rate-limiting enzyme, further aggravates HF after pressure overload (Wang et al., 2013). Apart from glycolysis, there are two other main pathways for glucose utilization: the pentose phosphate pathway (PPP) and the hexosamine biosynthesis pathway (HBP) (Tran and Wang, 2019). Glucose-6-phosphate dehydrogenase (G6PD), the rate-limiting enzyme of the PPP, is a protective mechanism in response to oxidative stress. HBP-regulated effects are found to be protective or maladaptive, depending on the observed conditions (Jain et al., 2003). Enhanced O-GlcNAcylation *via* the HBP is found to mediate protective effects in the diabetic heart (Marsh et al., 2011). However, transgenic overexpression of OGT with a consequence of altered O-GlcNAcylation is sufficient to alter cardiac function, whereas inhibition of O-GlcNAcylation by overexpression of protein O-GlcNAcase (OGA) protects from pressure overload induced cardiac malfunction (Umapathi et al., 2021). One of the proteins which is regulated by O-GlcNAcylation and may in part mediate adverse effects is the Ca^2+^-handling enzyme STIM1 (Lehmann et al., 2018). A regulating enzyme of the O-GlcNAcylation of STIM1, HDAC4, can be modified by O-GlcNAcylation itself. This phenomenon is identified to be a compensatory mechanism to prevent diabetes-associated HF (Kronlage et al., 2019). Given these data, metabolic disorders in FAO and glucose metabolism are part of a complex regulatory system and can be the cause as well as the consequence of HF.

In cancer, metabolism is principally shifting toward increased glucose utilization in response to a high demand of anabolic processes. Due to limited oxygen supply, this leads to high levels of lactate production. This phenomenon is known since the 1920s and named after his discoverer “Warburg” (Crabtree, 1928). Tumor suppressors and oncogenes are able to modulate the aerobic glycolysis of tumor cells and are thereby regulating tumor growth. P53 for example is able to downregulate phosphoglucomutase (PGM) and is thus limiting cancer cell survival (Kondoh et al., 2005).

Activation of Akt, which is linked to cardiac hypertrophy in cardiomyocytes, is maintaining a high glucose supply in cancer cells by upregulating Glucose transporter 1(GLUT-1) and hexokinase-1 (HK-1) (Barthel et al., 1999; Rathmell et al., 2003; Majewski et al., 2004). PGC-1α, a phosphorylation target of the serine/threonine kinase Akt, is a major regulator in cancer-associated metabolism as well (Li et al., 2007; Bost and Kaminski, 2019). PGC-1α is primarily responsible for maintaining the mitochondrial function including oxidative phosphorylation (Wu et al., 1999). This function is found to be intriguingly important in the survival of pancreatic cancer stem cells (Sancho et al., 2015). In breast cancer models, the Krebs cycle flux is dependent on PGC-1α, which itself depends on estrogen. In breast cancer patients, this metabolic activation is associated with a poor prognosis (McGuirk et al., 2013). Interestingly, cardiomyocyte-specific deletion of PGC-1α leads to peripartum-cardiomyopathy (PPCM), a hormone-sensitive cardiomyopathy in females after birth (Patten et al., 2012).

In prostate cancer, inhibition of PGC-1α reduces the androgen-receptor-dependent cell growth and might be used as a therapeutical approach (Shiota et al., 2010). The role of PGC-1α depends on the cancer type and the tissue-specific co-activating factor, such as the estrogen- or the androgen-receptor (Mastropasqua et al., 2018).

Increased flux in glycolysis is associated with tumor growth and upregulation of PFK (Chesney et al., 1999), but can occur in line with increased activity of the side-pathways, such as the HBP, as well. O-GlcNAcylation is a posttranslational modification which is linking metabolism to intracellular signaling in many tumor diseases (Ferrer et al., 2016). Exemplary, in breast cancer cell lines, the O-GlcNAcylation and the regulating enzyme, O-GlcNAc transferase (OGT), are found to regulate the transcription factor HIF1α and thereby GLUT-1 expression. Reduction of global O-GlcNAcylation leads to the induction of apoptosis (Ferrer et al., 2014). In breast cancer xenograft models with OGT deficiency, the tumor growth is reduced (Caldwell et al., 2010). In pancreas cancer cell lines, reduction of O-GlcNAcylation leads to the apoptosis as well. This effect is attributed to the transcription factor NFκB (Ma et al., 2013). Taken together, O-GlcNAcylation seems to be a central regulator in many tumor diseases, which can be used as a therapeutic target in cancer. In parallel, it plays an important role in cardiac function and dysregulation results in maladaptive response.

## Cardiac Metabolic Consequences of Cancer Therapies

The common metabolic alterations and signaling pathways in the heart and cancer might be influenced by cancer therapies as well (Table 3). Antiangiogenic drugs, for example, have been a milestone in the therapy of many progressed tumor diseases. Bevacizumab is the first vascular endothelial growth factor (VEGF) signaling pathway inhibitor to be approved by the FDA. It dramatically improves the progression-free survival of many tumor entities, such as colorectal carcinoma (McCormack and Keam, 2008). Tyrosine kinase inhibitors, also inhibiting angiogenesis and VEGF signaling, further improve cancer therapy (Shawver et al., 2002; Huang et al., 2020).

**TABLE 3:** Cancer therapies which influence cardiac metabolism.

**Group of drugs**	**Molecular target**	**Metabolic alterations in the heart**
VEGF inhibitors, e.g., Bevacizumab and tyrosine kinase inhibitors	GLUT-1 (Giordano et al., 2001), PGC-1α (Kivela et al., 2014), Akt (Kivela et al., 2014; Pandey et al., 2018)	• Arterial hypertension (Pandey et al., 2018) • Shift from FAO to glucose uptake in cardiomyocytes (Kivela et al., 2014)
Anthracyclines, e.g., doxorubicin	TopoIIβ (Zhang et al., 2012), PGC-1α (Zhang et al., 2012), IL-6 (Elsea et al., 2015)	• Increased apoptosis (Elsea et al., 2015; Willis et al., 2019) • Cachexia (Elsea et al., 2015; Willis et al., 2019) • Mitochondrial dysformation (Elsea et al., 2015)

*FAO, Fatty acid oxidation; GLUT-1, Glucose transporter 1; IL, Interleukin; PGC-1α, Peroxisome proliferator-activated receptor-gamma coactivator; Akt, Protein kinase B; TopoIIβ, Topoisomerase IIβ; VEGF, Vascular endothelial growth factor.*

A cardiomyocyte-specific KO mouse of VEGF shows reduced microvasculature, reduced ejection fraction and the induction of hypoxia-induced genes, including GLUT-1 (Giordano et al., 2001). In VEGF transgenic rats, there is a hypervascularization of the heart and a metabolic shift from FAO to glucose metabolism. Among others, those changes are linked to an altered expression of PGC-1α and an activation of the Akt-signaling pathway (Kivela et al., 2014). Thus, angiogenesis inhibitors may contribute to cardiotoxic and specifically metabolic alterations. Commonly, arterial hypertension is a known adverse effect of VEGF inhibitors. This in turn may activate kinases, such as Akt (Pandey et al., 2018).

Anthracyclines, e.g., doxorubicin, have been used for cancer therapy for over 60 years and still are included in standard chemotherapy regimens (Rivankar, 2014). Cardiotoxic effects of doxorubicin depend on topoisomerase IIβ (TopoIIβ), which is shown in TopoIIβ KO mice. Interestingly, one of the downstream targets of this TopoIIβ-dependent effect is PGC-1α (Zhang et al., 2012). The animals show structural changes of the mitochondria, indicating a similar metabolic change compared to primary HF. A doxorubicin-containing chemotherapy induces IL-6 release in non-tumor bearing mice and is associated with loss of lean body mass, comparable to other IL-6 tumor models (Elsea et al., 2015). Data from patients support the notion of a pro-atrophic effect of anthracyclines (Willis et al., 2019).

## Discussion and Conclusion

Cancer and cardiovascular diseases are two major burdens in the treatment of an increasing number of patients. In this review, we focus on the interaction of both diseases and their relation to metabolic alterations. Both, cancer and HF, are associated with malnutrition and cachexia. A significantly increased mortality is the clinical consequence of these important co-morbidities.

**FIGURE 1:**
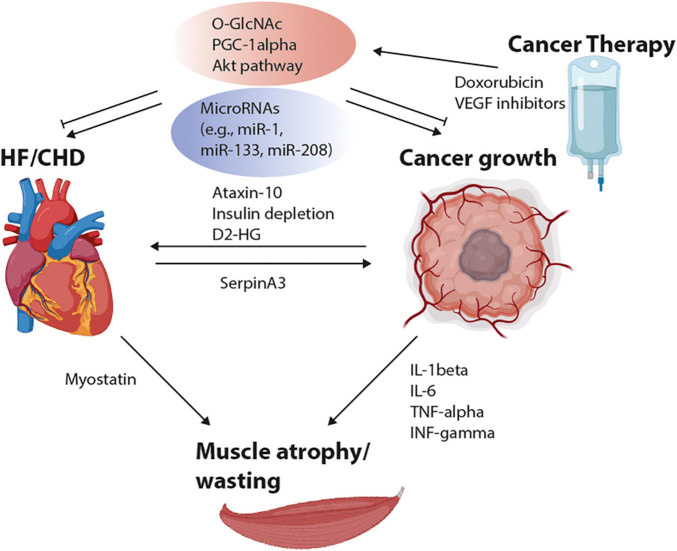
Scheme of interacting factors between heart failure (HF), coronary heart disease (CHD), cancer growth, and muscle atrophy. Cytokine release from the tumor (Ataxin-10, D2-HG, IL-1/6, TNF-α, and INF-γ) may influence cardiac function and muscle wasting. Released factors from the heart, such as Myostatin and SerpinA3 are able to regulate muscular and cancer growth. Alterations in microRNAs (miRs) and metabolic pathways, including the hexosamine biosynthesis pathways (HBPs), PGC-1α and Akt activation are regulating cardiac remodeling as well as tumor growth. The adaptations in metabolic signaling pathways may be influenced by chemotherapies, e.g., by VEGF inhibitors and doxorubicin. D2-HG, D-2-hydroxyglurate; INF-γ, Interferon-gamma; IL, Interleukin; PGC-1α, Peroxisome proliferator-activated receptor-gamma coactivator; Akt, Protein kinase B; TNF-α, Tumor necrosis factor alpha; VEGF, Vascular endothelial growth factor. Created with Biorender.com.

Looking at different preclinical studies, we observe multiple factors which are influencing tumor growth, cachexia, HF and intracellular substrate utilization. The mediators are summarized in Table 1. Inflammation and especially cytokines, possibly derived from malignant or cardiovascular diseases, are important mediators of muscle atrophy. Other primary non-inflammatory cytokines can be excreted from the heart, such as myostatin or SerpinA3, or from the tumor, such as Ataxin-10. These cytokines can lead to either tumor growth or wasting. Besides, several miRs (miR-1, miR-133, miR-145, miR-208) are linked to cardiac remodeling and tumor growth. In addition, FAO- and glycolysis-regulating enzymes, e.g., PGC-1α, HK-1, GLUT-1, as well as side pathways of glycolysis (PPP, HBP) are linked to HF and tumor growth, respectively (Figure 1).

These studies suggest many concurrent molecular alterations of both diseases, which might explain the co-occurrence of these diseases. Further, cancer therapies, e.g., angiogenesis inhibitors and anthracyclines, may also intervene at the same intracellular metabolic targets.

Future oncological and cardiological therapies should keep these interdisciplinary effects in mind, in order to minimize adverse effects and reduce mortality and morbidity.

The knowledge of the specific molecular alterations may lead to personalized treatment options in cancer therapies and to personalized cardiac surveillance protocols, according to cancer type and previous metabolomic profiles.

## Author Contributions

DF, MH, NF, and LHL contributed to the conception and design of the review. DF wrote the first draft of the manuscript. DF and LHL wrote sections of the manuscript. All authors contributed to manuscript revision and approved the submitted version.

## Conflict of Interest

LHL has served on the advisory board for Daiichi Sankyo, Senaca, and Servier, as well as an external expert for Astra Zeneca and received speakers’ honoraria from Novartis and MSD. The remaining authors declare that the research was conducted in the absence of any commercial or financial relationships that could be construed as a potential conflict of interest.

## Publisher’s Note

All claims expressed in this article are solely those of the authors and do not necessarily represent those of their affiliated organizations, or those of the publisher, the editors and the reviewers. Any product that may be evaluated in this article, or claim that may be made by its manufacturer, is not guaranteed or endorsed by the publisher.
